# Fucoidan Induces Cancer Cell Apoptosis by Modulating the Endoplasmic Reticulum Stress Cascades

**DOI:** 10.1371/journal.pone.0108157

**Published:** 2014-09-18

**Authors:** Shaohua Chen, Yang Zhao, Yu Zhang, Daohai Zhang

**Affiliations:** 1 Department of Gastroenterology, The First Affiliated Hospital, Zhejiang University, Hangzhou, P. R. China; 2 Centenary Institute of Cancer Medicine and Cell Biology, University of Sydney, Sydney, New South Wales, Australia; 3 Department of Oncology, Zhejiang Hospital, Hangzhou, P. R. China; 4 Caner Research Group, The Canberra Hospital, ANU Medical School, The Australia National University, Canberra, Australia; Taipei Medicine University, Taiwan

## Abstract

**Background:**

Cancer metastasis is the main cause leading to disease recurrence and high mortality in cancer patients. Therefore, inhibiting metastasis process or killing metastatic cancer cells by inducing apoptosis is of clinical importance in improving cancer patient survival. Previous studies revealed that fucoidan, a fucose-rich polysaccharide isolated from marine brown alga, is a promising natural product with significant anti-cancer activity. However, little is known about the role of endoplasmic reticulum (ER) stress in fucoidan-induced cell apoptosis.

**Principal Findings:**

We reported that fucoidan treatment inhibits cell growth and induces apoptosis in cancer cells. Fucoidan treatments resulted in down-regulation of the glucose regulated protein 78 (GRP78) in the metastatic MDA-MB-231 breast cancer cells, and of the ER protein 29 (ERp29) in the metastatic HCT116 colon cancer cells. However, fucoidan treatment promoted ER Ca^2+^-dependent calmodulin-dependent kinase II (CaMKII) phosphorylation, Bcl-associated X protein (Bax) and caspase 12 expression in MDA-MB-231 cells, but not in HCT116 cells. In both types of cancer cells, fucoidan activated the phosphorylation of eukaryotic initiation factor 2 alpha (p-eIF2α)\CCAAT/enhancer binding protein homologous protein (CHOP) pro-apoptotic cascade and inhibited the phosphorylation of inositol-requiring kinase 1 (p-IRE-1)\X-box binding proteins 1 splicing (XBP-1s) pro-survival cascade. Furthermore, CHOP knockdown prevented DNA damage and cell death induced by fucoidan.

**Conclusion/Significance:**

Fucoidan exerts its anti-tumor function by modulating ER stress cascades. Contribution of ER stress to the fucoidan-induced cell apoptosis augments our understanding of the molecular mechanisms underlying its anti-tumour activity and provides evidence for the therapeutic application of fucoidan in cancer.

## Introduction

Cancer is a chronic disease with high mortality due to its high metastatic ability and resistance to chemo- and radio-therapy. Despite the sophisticates of therapeutic strategy for cancer treatment, no treatment is 100% effective against disseminated/metastatic cancer. Until recently, most of the therapeutic drugs target on the proliferative cancer cells for the treatment of primary tumours. Given that most cancer deaths are the result of metastatic disease, understanding the mechanisms of cancer metastasis and developing drugs for metastatic cancer are indeed emerging areas in cancer cell biology and cancer therapy.

Developing natural products for cancer therapy is a promising strategy for cancer treatment and prevention. For instance, fucoidan, a fucose-rich polysaccharide, is isolated from brown seaweed such *Cladosiphon okamuranus* and *Fucus evanescens* [1], [2]. Fucoidan is structurally similar to heparin, with a substantial percentage of L-fucose [3], [4]. Recent studies have demonstrated its various biological activities including anti-inflammatory, anti-coagulant [5], anti-HIV and anti-cancer [6]–[9] activities. Significantly, fucoidan shows a high efficiency in the treatment of a variety of cancers, including breast cancer, prostate cancer, lung cancer, hepatoma and leukemia [7], [8], [10]–[12]. Its anti-tumour activity is exerted by regulating multiple signalling pathways in cancer cells. It was found that fucoidan can induce cell apoptosis *via* the activation of caspase-cascades, extracellular signal-regulated kinase mitogen-activated protein kinase (ERK1/2 MAPK) and the inactivation of p38MAPK and phosphatidylinositol 3-kinase (PI3 K)/protein kinase B (Akt) [7], [11], [13]. In addition, fucoidan also inhibits Wnt/β-catenin pathway to decrease cyclin D1 expression, leading to cell cycle arrest *in vitro* and *in vivo* [7], [14]. The *in vivo* studies demonstrated that fucoidan suppressed tumour growth and significantly diminished lung metastasis of 4T1 breast cancer cells [14]–[16]. Collectively, these results support the potential development of fucoidan as an anticancer drug. Albeit this, the mechanisms of action that fucoidan exerts on cancer cell apoptosis have not been fully understood. In particular, little is known about the involvement of endoplasmic reticulum (ER) stress, a central signalling that defines cell’s fate, in the fucoidan-mediated anti-tumour activity.

ER plays a crucial role in Ca^2+^ homeostasis and cell pathophysiology. Accumulation of unfolded or misfolded proteins within the ER or Ca^2+^ store depletion induces ER stress and triggers the unfolded protein response to maintain ER homeostasis [17]. Under resting conditions, the ER chaperone protein, the glucose regulated protein 78 (GRP78), seals the pore of the translocon in the ER and thus, reduces ER Ca^2+^ leak [18]. Under ER stress, GRP78 is released from the translocon and triggers ER Ca^2+^ depletion [19]. Cytosolic Ca^2+^ binds to calmodulin to activate Ca^2+^\calmodulin-dependent kinase II (CaMKII) signalling, leading to ER stress-induced cell apoptosis through activating the mitochondrial apoptosis pathway [20].

ER stress also leads to dissociation of GRP78 from the complexes formed with the luminal part of ER membrane proteins, protein kinase RNA (PKR)-like ER kinase (PERK), inositol-requiring kinase 1 (IRE1) and activating transcription factor 6 (ATF6), resulting in autophosphorylation of PERK and IRE-1 and translocation of ATF6 to the Golgi for cleavage [21]. These alterations cause activation of their downstream signalling pathways. For instance, the activated PERK phosphorylates eukaryotic initiation factor 2 alpha (eIF2α) to attenuate protein translation and reduce ER protein overload [22]. Prolonged ER stress also induces ATF4 and CCAAT/enhancer binding protein homologous protein (CHOP) expression, leading to apoptosis [17]. To cope with ER stress, activated IRE-1 acts as an endonuclease to increase the X-box binding protein 1 (XBP-1) splicing, thereby leading to upregulation of genes important for cell survival during ER stress [23]. ATF6 activation occurs after proteolytic cleavage in the Golgi, followed by its nuclear translocation for the adaptive stress response [24]. To some extent, ER stress plays a crucial role in tuning these signalling balances to manipulate the cell’s fate [17].

In this study, we investigated whether ER stress involves the fucoidan-induced cell apoptosis in the highly invasive and metastatic MDA-MB-231 breast cancer cells [25] and HCT116 colon cancer cells [26]. We showed that fucoidan treatment regulates the ER Ca^2+^-modulated phosphorylation of CaMKII in a cell type-dependent manner. However, fucoidan treatment in both types of cancer cells activated the p-eIF2\CHOP pro-apoptotic cascade and inhibited the p-IRE-1/XBP-1s pro-survival cascade. Therefore, ER stress contributes, at least in part, to fucoidan-induced cell apoptosis.

## Materials and Methods

### Cell culture

Human MDA-MB-231 breast cancer cells and HCT116 colon cancer cells were purchased from the American Type Culture Collection (ATCC, Manassas, VA) and cultured in Dulbecco’s Modified Eagle’s Medium (DMEM) supplemented with 10% fetal bovine serum (FBS, Invitrogen, Eugene, OR). Cells were cultured at 37°C with 5% CO_2_ in a humidified incubator.

### Antibodies and reagents

The following antibodies were used in this study: rabbit anti-GRP78 and rabbit anti-ERp29 from Novus Biologicals (Littleton, CO); rabbit anti-eIF2a, mouse anti-phospho-eIF2a (S51), rabbit anti-p58^IPK^, rabbit anti-cleaved PARP, rabbit anti-spliced XBP-1 (XBP-1s), rabbit anti-cleaved caspase 3and mouse anti-GADD153/CHOP from Cell Signalling Technology (Beverly, MA); mouse anti-β-actin from Sigma-Aldrich (St Louis, MO); rabbit anti-CaMKII, rabbit anti-Bax, rabbit anti-caspase 12 and rabbit anti-phospho-CaMKII (T286) from Abcam (Cambridge, MA).

Fucoidan isolated from *Fucus vesiculosus* was purchased from Sigma-Aldrich **(**Saint Louis, MO). The complete, EDTA-free protease inhibitor cocktail tablets were obtained from Roche Diagnostics (Indianapolis, IN). Phosphatase cocktail inhibitors I and II and thapsigargin (TG) were from Sigma-Aldrich (Steinheim, Germany). Lipofectamine 2000 transfection reagents were supplied from Invitrogen (Eugene, OR). Salubrinal was purchased from Tocris Bioscence (Ellisville, MO).

### Cell growth and viability

Cell growth was assessed using the CellTiter96 AQueous One Solution Cell Proliferation Assay (Promega, Madison, WI). In brief, cells were seeded in triplicate in 96-well plates at a density of 5000 cells per well in 100 µl of the corresponding medium. On the following day, the media were aspirated and replaced with fresh media containing the indicated concentrations of fucoidan (0, 10, 50, and 100 µg/ml). Cells were incubated under standard culture conditions for up to 4 days, and viable cells were assessed. The absorbance at 492 nm was measured using an Infinite F200 microplate reader (TECAN Austria GmbH, Grodig, Austria). Triplicate experiments were performed for each treatment. Cell viability was also examined using the trypan blue exclusion assay.

### Cell apoptosis

Cell apoptosis was assayed using terminal deoxynucleotidyl transferase-mediated dUTP nick end labelling (TUNEL) assay [27]. Briefly, cells (2.5×10^4^ per well) were seeded in 6-well culture plates with coverslip and 24 h later, 2 ml of fresh medium with fucoidan (final conc. 100 µg/ml) was added to each well. Cells were then treated for 3 days followed by TUNEL staining using the DeadEnd Fluorometric TUNEL system kit (Promega, Madison, WI) according to the manufacturer’s protocol. The slides were mounted using antifade mounting fluid containing DAPI. Green (apoptotic cell nucleus) and blue (DAPI, total cells) fluorescent signals were captured using an Olympus Fluoview FV1000 confocal laser scanning microscope (Olympus, Japan). The percentage of apoptotic cells was expressed as an average of five randomly selected fields (300 cells per field). In addition, cell apoptosis was also examined by immunoblotting the expression of cleaved caspase 3 and cleaved Poly (ADP-ribose) polymerase (PARP).

### RNA interference

Small interfering RNAs (siRNAs) targeting CHOP (CHOP siGENOME SMARTpool, Dharmacon, Lafayette, CO) and siGENOME non-targeting siRNA were used for CHOP knockdown and control, respectively. Briefly, cells were grown in 6-well plates and transiently transfected at 60–80% confluence with siRNA at a final concentration of 25 nM using Lipofectamine 2000 transfection reagent (Invitrogen, Eugene, OR) according to the manufacturer’s instructions. Forty eight hours after transfection, the cells were incubated with fresh medium alone or medium with 100 µg/ml of fucoidan. After 3 days post-treatment, cells were harvested for analysing viability using trypan blue exclusion assay and assessing the levels of CHOP and cleaved PARP by immunoblot.

### Immunoblot analysis

Cells at 70–80% confluence were treated with fucoidan at various concentrations (0, 1.0, 5.0, 10, 50 and 100 µg/ml, respectively) for 3 days. Both the attached and suspended cells were harvested for protein extraction. Cell lysates were extracted with RIPA Buffer (1% Igepal, 1% sodium deoxycholate, 0.15 M sodium chloride, 0.01 M sodium sulphate, pH 7.2 and 2 mM EDTA), supplemented with protease inhibitors (Roche Diagnostics) and phosphatase cocktail inhibitors I and II (1∶100, Sigma-Aldrich). Western blotting was carried out as described [28]. Briefly, total proteins (30 µg/lane) were separated by 10% SDS-PAGE and transferred onto PVDF membranes. Membranes were blocked with 5% skim milk in Tris-buffered saline buffer with 0.1% Tween 20 for 1 h at room temperature and probed with the indicated primary antibodies. Goat anti-mouse horseradish peroxidase (HRP; Upstate Biotechnology, Lake Placid, NY) or goat anti-rabbit HRP secondary antibody (ZyMED Laboratories, San Francisco, CA) was used as secondary antibodies. The chemiluminescent signal was developed with Supersignal West Pico Chemiluminescent Substrate (Pierce, Rockford, IL). Signal intensity was analysed using GeneTools software (Syngene, Frederick, MD). The level of β-actin was used as a loading control.

### Statistical analyses

One-way analysis of variance (ANOVA) and Student’s *t* tests were used to analyse the significance of differences. p<0.05 was considered as significant. All cell culture experiments were performed in triplicate. Data are presented as mean ± standard deviation (SD) and supplied in Table S1.

## Results

### Fucoidan treatment induces cell apoptosis

**Figure 1:**
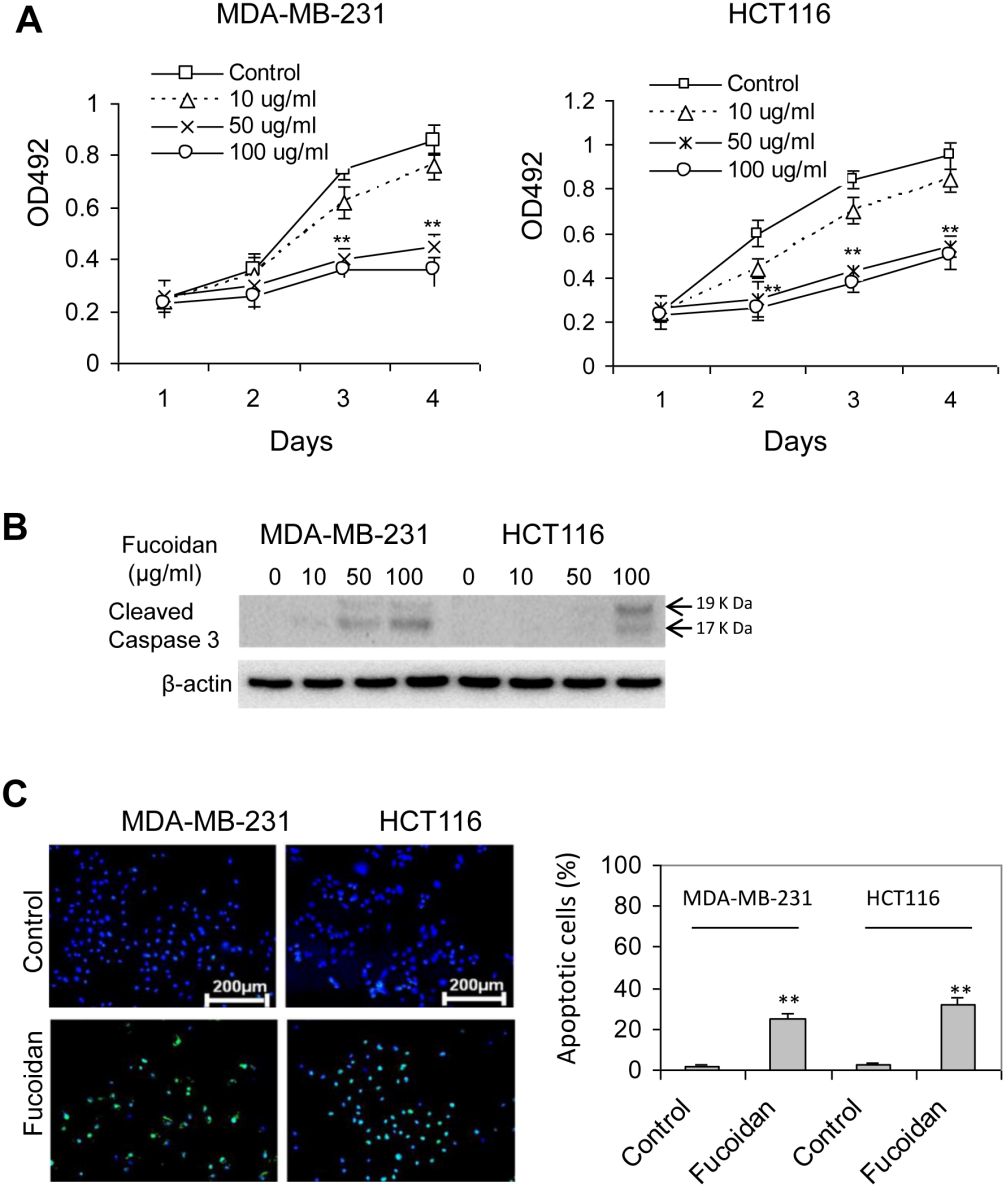
Fucoidan inhibits cell growth and promotes cell apoptosis. (A) Cell growth. Cells were treated with fucoidan at the indicated concentration and the viable cells were assessed using the CellTiter96 AQueous One Solution Cell Proliferation Assay. (B) Caspase 3 activation. Cells were treated with fucoidan for 3 days and the expression of cleaved caspase 3 was examined by Western blot. (C) TUNEL. For TUNEL analysis, cells were treated with fucoidan at 100 µg/ml for 3 days and the cell apoptosis was analysed using TUNEL assay. Apoptotic cells (green) were counted in five randomly selected fields (300 cells per field) and presented as a mean ± SD of percentage of apoptotic cells over the total cells (DAPI, blue). Data are expressed as a mean ± SD in triplicate experiments. *p<0.05; **p<0.01.

Previous studies demonstrated a significant inhibition of fucidan on tumorigenesis and cancer cell metastasis [14]–[16]. To understand the effect of fucoidan on cell growth and apoptosis, both the metastatic MDA-MB-231 cells and HCT116 cells were treated with fucoidan at the indicated concentration for up to 4 days and the cell growth was assayed. As shown in Fig. 1A, cell growth was significantly inhibited when the cells were treated with >50 µg/ml fucoidan on day 3 for MDA-MB-231 cells and on day 2 for HCT116 cells, compared to their respective control cells. In addition, to determine whether cell apoptosis attributes to the inhibition of cell growth, both types of cells were treated with fucoidan (100 µg/ml) for 3 days and cell apoptosis was assessed by examining the expression of cleaved caspase 3 and TUNEL. As indicated in Figure 1B, treatment of fucoidan at high doses (*e.g*., 100 µg/ml) caused high expression of cleaved caspase 3 in both types of cells. TUNEL analysis also showed an approximately >25% cells undergoing DNA damage in fucoidan-treated cells (Fig. 1C). Hence, fucoidan treatment resulted in remarkable DNA damage in these cells.

### Fucoidan treatment leads to different ER response in MDA-MB-231 and HCT116 cells

**Figure 2:**
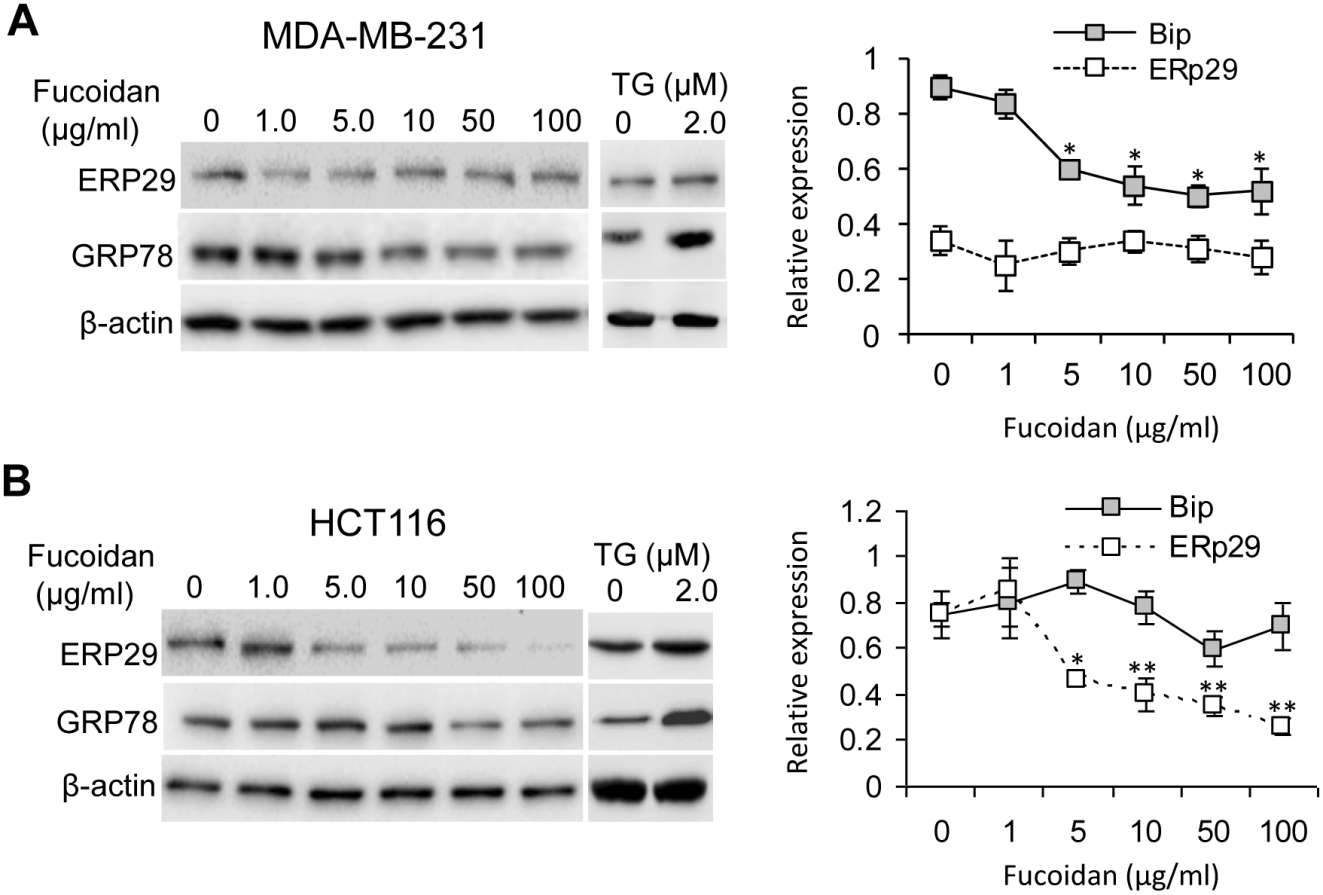
Effect of fucoidan on the expression GRP78 and ERp29. Cells at 70–80% confluence were treated with fucoidan at the indicated concentrations for 3 days and both the attached and suspended cells were harvested for protein expression analysis. (A) The expression of GRP78, but not ERp29, was inhibited by fucoidan in MDA-MB-231 cells; (B) The expression of ERp29 was significantly attenuated by fucoidan in HCT116 cells, while GRP78 was only slightly decreased (∼1.5-fold) in cells treated with fucoidan at >50 µg/ml. As a positive control, cells were treated with ER stress inducer TG (2.0 µM) for 24 h. Data represent mean ± SD in triplicate experiments. *p<0.05; **p<0.01.

To investigate whether ER stress is involved in fucoidan-induced cell apoptosis, we initially examined the expression of ER stress markers including GRP78 and ERp29. Interestingly, contrary to the cells treated with ER stress inducer TG,, the expression of GRP78 was decreased in the fucoidan-treated MDA-MB-231 cells with no effect on ERp29 expression in these cells, relative to the control cells (no fucoidan treatment) (Fig. 2A). Instead, the expression of ERp29 was significantly reduced in the fucoidan-treated HCT116 cells, whereas the expression of GRP78 was slightly increased when the cells were treated with 5 µg/ml, followed by reduction with high doses of treatments (Fig. 2B). GRP78 has multiple functions in cancer cells such as being responsible for cell proliferation, protecting cells from apoptosis and accelerating ER-associated protein degradation of misfolded proteins [29]. Recent studies have also addressed the role of ERp29 in cancer cell survival against chemo- and radio-therapy [27], [30]. The reduction of GRP78 or ERp29 in the fucoidan-treated cells suggests that fucoidan induces cell death by suppressing the expression of these survival proteins in MDA-MB-231 and HCT116 cells.

### Fucoidan treatment results in activation of p-CAMKII\Bax and caspase 12 expression in a cell-context dependent manner

**Figure 3:**
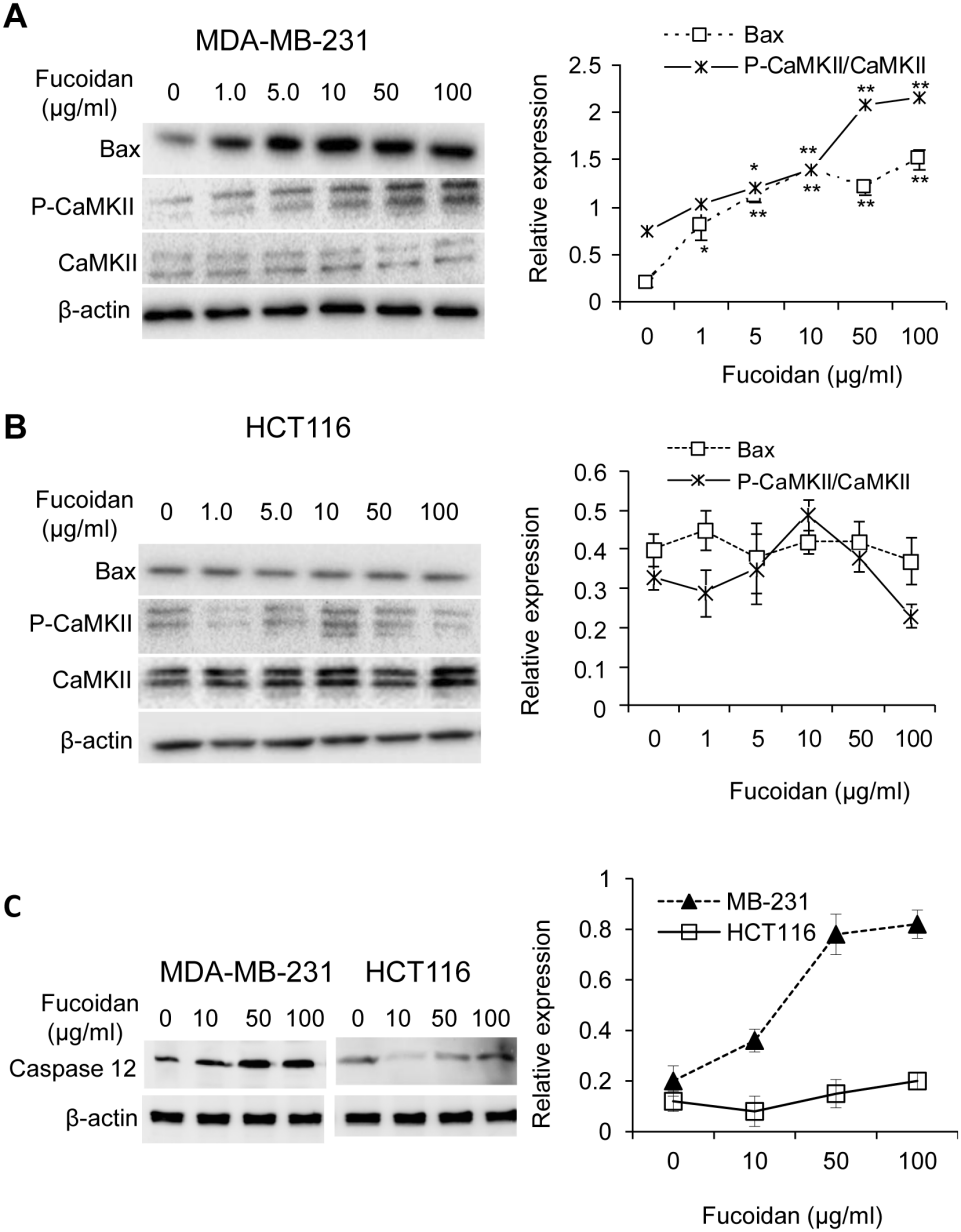
Effect of fucoidan on the relative phosphorylation of CaMKII (p-CaMKII/CaMKII) and Bax expression in MDA-MB-231 cells (A) and HCT116 cells (B). (A) Relative phosphorylation of CaMKII and Bax expression were progressively increased by fucoidan in MDA-MB-231 cells; (B) Relative phosphorylation of CaMKII was moderately stimulated (∼1.5-fold) at 10 µg/ml, followed by inhibition (∼1.4-fold) of fucoidan at 100 µg/ml. Bax expression was not affected by fucoidan in HCT116 cells. (C) Caspase 12 expression and cleavage. Fucoidan efficiently induces caspase 12 expression in MDA-MB-231 cells, rather than in HCT116 cells. No cleavage of caspase 12 was found in both types of cells. Data represent mean ± SD from triplicate experiments. *p<0.05; **p<0.01.

Because GRP78 can bind with the translocon in the ER membrane to block Ca^2+^ release into the cytosol [18], we next analyzed whether reduction of GRP78 in MDA-MB-231 cells could induce Ca^2+^-mediated activation of CaMKII and the expression of apoptosis-related proteins Bax. As shown in Fig. 3A, the relative phosphorylation of CaMKII (p-CaNKII/CaMKII) was progressively increased in the cells treated with fucoidan compared to the control cells (no fucoidan treatment), indicating an activation of Ca^2+^\CaMKII signalling. In line with this, Bax expression was also significantly increased and maintained at a similar level in the cells treated with fucoidan (10–100 µg/ml). In contrast, comparing to the control cells, the relative phosphorylation of CaMKII (p-CaNKII/CaMKII) was moderately increased (∼1.5-fold) in HCT116 cells treated with 10 µg/ml of fucoidan, followed by reduction in those cells treated with 100 µg/ml of fucoidan. Overall, fucoidan treatment in HCT116 cells did not cause significant change of the relative phosphorylation of CaMKII and Bax expression (Fig. 3B). We also found that the expression of caspase 12 was highly increased with fucoidan concentration in MDA-MB-231 cells, but not in HCT116 cells (Fig. 3C). In addition, no cleavage of caspase 12 was observed when probing with antibody. Taken together, these data suggest that fucoidan regulates Ca^2+^\ CaMKII signalling and caspase 12 in a cell type-dependent manner.

### Fucoidan treatment inhibits p-IRE-1\XBP-1 splicing

**Figure 4:**
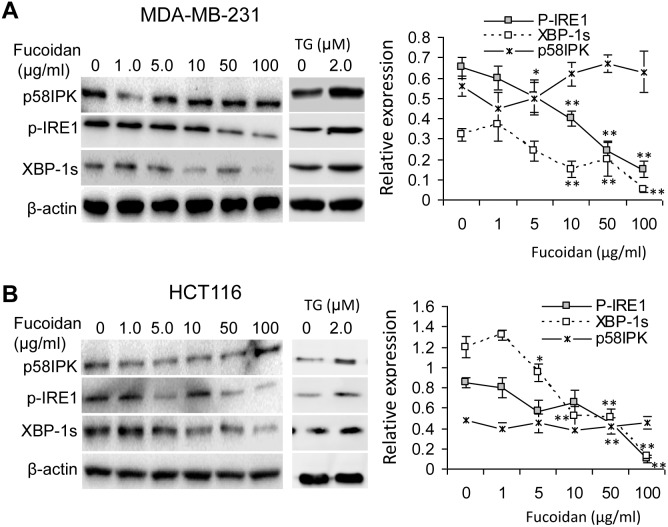
Fucoidan suppresses p-IRE-1\XBP-1 splicing in MDA-MB-231 cells (A) and HCT116 cells (B). Cells were treated with fucoidan as described in the “Materials and Methods” and in Figure 2. The phosphorylation of IRE-1 (p-IRE-1) and XBP-1 splicing were remarkably reduced by fucoidan as assessed by immunoblot. Its downstream target, p58^IPK^, was not subsequently decreased. Data are expressed as mean ± SD in triplicate experiments. Note that TG-treated cells (2.0 µM, 24 h) showed an increased p-IRE-1 and XBP-1s in both cells. *p<0.05; **p<0.01.

Based on the above findings that fucoidan can modulate ER stress response in the cancer cells, we next examined whether fucoidan differentially regulates IRE-1\XBP-1 s, a cascade defining cell survival response *via* up-regulating the expression of chaperones for ER folding capacity [31], and the PERK\P-eIF2α\CHOP pro-apoptotic cascade [17]. Compared to the control, fucoidan significantly inhibited the expression of XBP-1s in both types of cells (Fig. 4A, B). This was caused by the decreased phosphorylation of IRE-1 in the fucoidan-treated cells, because the activated p-IRE-1 functions as an endonuclease to generate spliced XBP-1s from unspliced XBP-1 mRNA under ER stress [32]. In contrast to the TG-treated cells demonstrating enhanced IRE-1 phosphorylation and XBP-1s expression (Fig. 4A, B), these results indicate an inhibitory effect of fucoidan on ER stress-related cell survival cascade. However, we did not observe a significant change of P58^IPK^, one of the downstream targets of XBP-1s and ATF6 [23], in fucoidan-treated cells.

### Fucoidan activates eIF2α phosphorylation and upregulates CHOP expression

**Figure 5:**
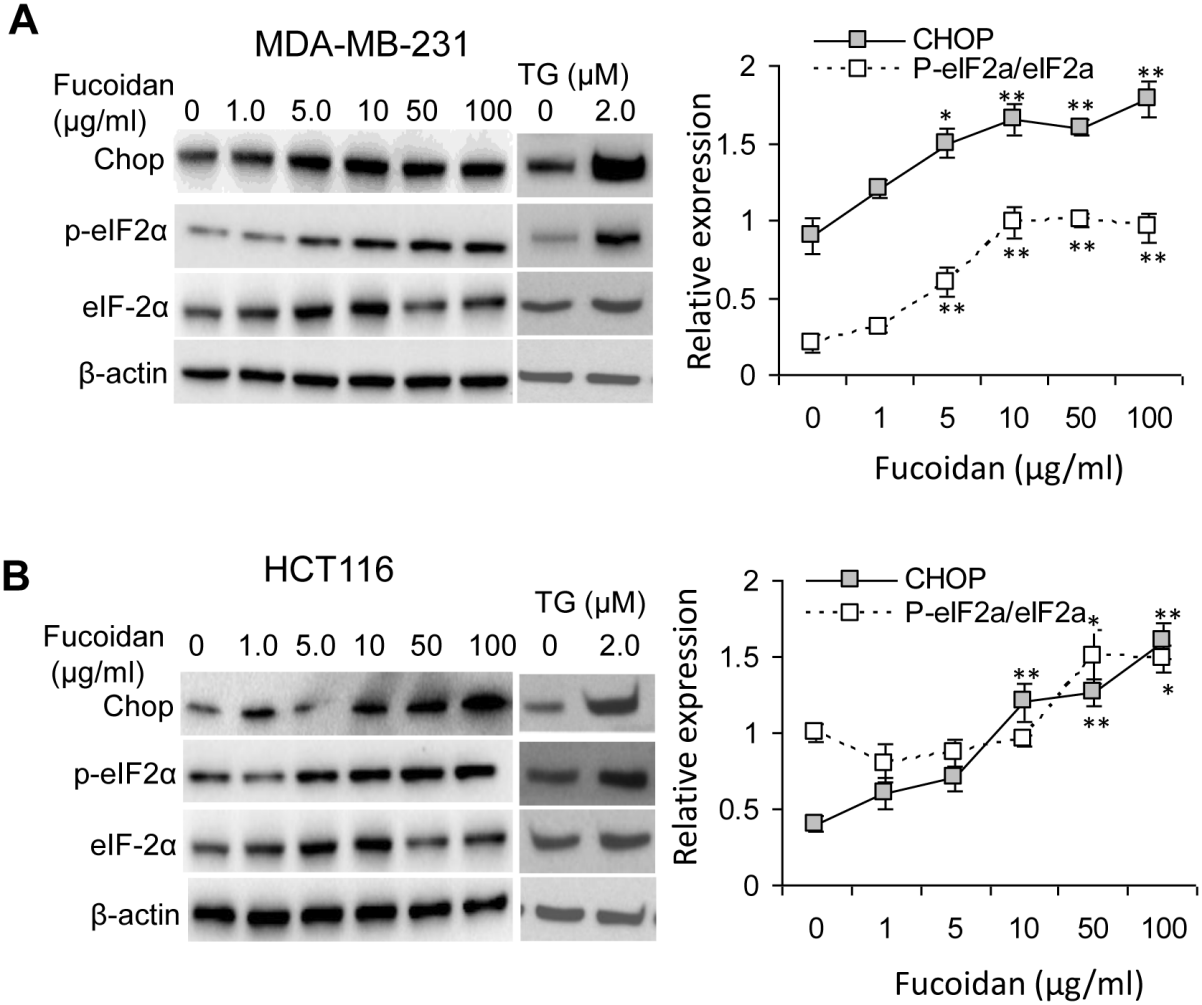
Fucoidan activates p-eIF2α\CHOP in MDA-MB-231 cells (A) and HCT116 cells (B). Cells were treated with fucoidan as described in the “Materials and Methods” and in Figure 2. The relative phosphorylation of eIF2α (p-eIF2α/eIF2α) and CHOP expression were remarkably increased by fucoidan as assessed by immunoblot. TG treatment (2.0 µM, 24 h) induced activation of p-eIF2α and CHOP expression. Data are expressed as mean ± SD in triplicate experiments. *p<0.05; **p<0.01.

Phosphorylation of eIF2α is a classical mechanism for down-regulating global protein synthesis to cope with ER stress [33]. Nevertheless, excessive ER stress also promotes cell death *via* upregulating ATF4\CHOP cascade [17]. It is thus speculated that this cascade is probably activated to participate in the fucoidan-induced cell apoptosis. Indeed, consistent with the TG-treated cells, the phosphorylation of eIF2α and expression of CHOP were progressively induced in a dose-dependent manner in both types of cells (Fig. 5A & B). Therefore, fucoidan can induce ER stress-related pro-apoptotic cascade in these cancer cells.

### Salubrinal treatment enhances fucoidan-activated eIF2α phosphorylation and CHOP expression

**Figure 6:**
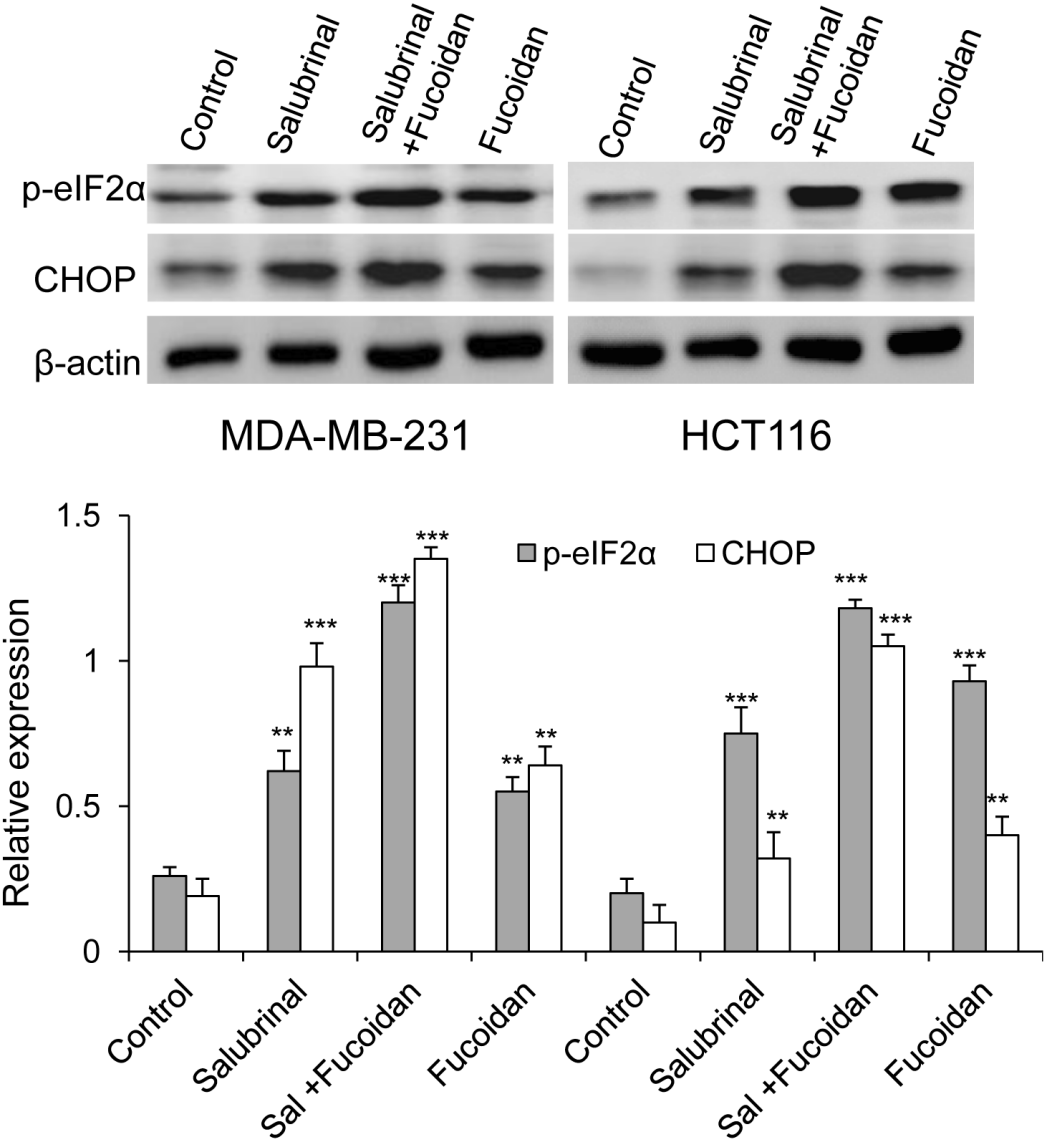
Combinatory treatment of fucoidan and salubrinal enhances eIF2α phosphorylation and CHOP expression in MDA-MB-231 cells and HCT116 cells. Cells were treated with salubrinal (50 µM) alone or in combination with fucoidan (100 µg/ml) for 48 h. Cell lysates (30 µg) were applied for the analysis of the expression of p-eIF2α and CHOP by immunoblot. **p<0.01; ***p<0.001.

Salubrinal is a selective inhibitor of eIF2α dephosphorylation and induces hyperphosphorylation of eIF2α by inhibiting GADD34-PP1C complex [34], [35]. To assess whether salubrinal treatment facilitates fucoidan-induced ER stress cascade, MDA-MB-231 and HCT116 cells were treated with salubrinal (50 µM) alone or in combination with fucoidan (100 µg/ml) for 48 hours and the expression of p-eIF2α and CHOP was examined. As indicated in Figure 6, cells treated with salubrinal and fucoidan showed higher expression of p-eIF2α phosphorylation and CHOP than the cells treated with salubrinal or fucoidan alone. These data indicate a stimulative effect of fucoidan on the salubrinal-induced p- eIF2α \CHOP cascade.

### CHOP silence inhibits fucoidan-induced expression of cleaved PARP

**Figure 7:**
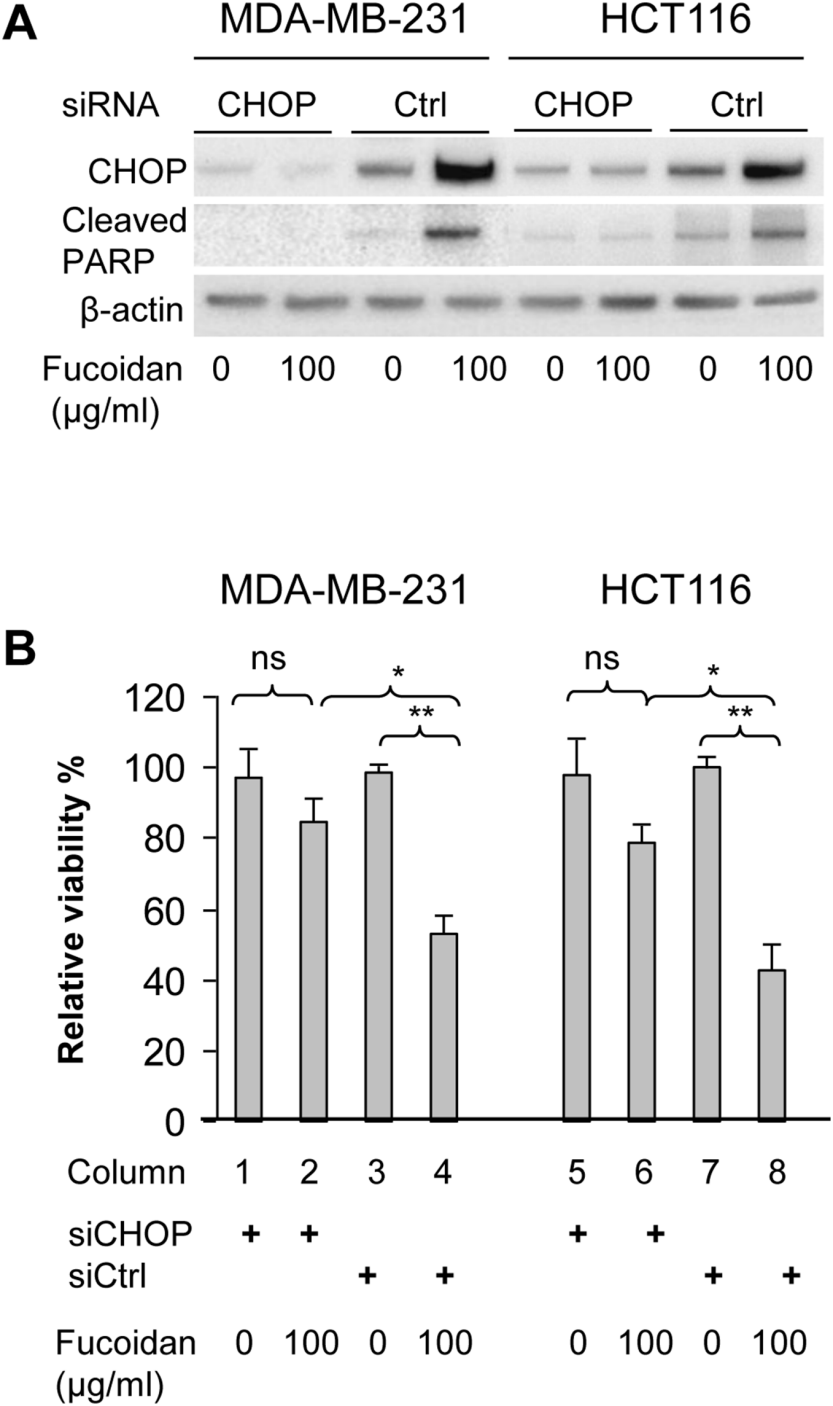
Effect of silencing CHOP on fucoidan-induced DNA damage and cytotoxicity. (A) CHOP knockdown by siRNA inhibits fucoidan-induced expression of cleaved PARP. Cells at 70–80% confluence were treated with CHOP siRNA or scramble siRNA for 48 h, followed by fucoidan treatment (0 or 100 µg/ml) for 2 days. The levels of CHOP and cleaved PARP were assessed by immunoblot. (B) Silencing CHOP antagonizes fucoidan-induced cell death. Cells pre-treated with CHOP siRNA or control siRNA were treated with fucoidan (0 or 100 µg/ml) for 2 days and the cell viability was examined using the trypan blue exclusion assay. Cell viability was expressed as a percentage of the siRNA control cells without fucoidan treatment (column 3 in MDA-MB-231 cells; column 7 in HCT116 cells). Note that CHOP knockdown alone does not affect cell viability in both types of cells (column 1 *vs.* 3; column 5 *vs.* 7). Instead, CHOP knockdown remarkably prevents cell apoptosis induced by fucoidan at 100 µg/ml (column 2 *vs.* 4 in MDA-MB-231 cells; column 6 *vs.* 8 in HCT116 cells). Results are interpreted as mean (±SD) of triplicate experiments. *p<0.05; **p<0.01.

To assess whether the fucoidan-induced CHOP in both types of cancer cells is linked to the observed cell apoptosis, cells were treated with siRNA targeting CHOP to reduce its endogenous levels or treated with scramble siRNA as a control. Our preliminary studies showed that both types of cells treated with 25 nM siRNA for 48 h could lead to >70% repression of CHOP expression compared to those treated with control siRNA. These cells were then treated with fucoidan (at 0 or 100 µg/ml) for 2 days and CHOP expression and DNA damage (as assessed by cleaved PARP) were analyzed. As shown in Fig. 7A, the level of CHOP was reduced by 70–90% of the controls in both types of cells after treatment. In the cells pre-treated with siRNA control, fucoidan treatment markedly increased CHOP expression and PARP cleavage over the cells with no fucoidan treatment. In contrast, fucoidan treatment was unable to induce CHOP expression and PARP cleavage in the CHOP-silenced cells, indicating that silencing CHOP is able to block fucoidan-induced expression of cleaved PARP. These data provide evidence that CHOP participates in fucoidan-induced DNA damage.

### CHOP repression antagonises fucoidan-induced cell apoptosis

To examine the effect of CHOP knockdown on the fucoidan-induced cell apoptosis, cell viability of these treated cells was examined. Relative to the cell viability of the siRNA control cells without fucoidan treatment (Fig. 7B, column 3 in MDA-MB-231 cells; column 7 in HCT116 cells), the cell viability in the CHOP-silenced cells was similar to that observed in these control cells (Fig. 7B, column 1 *vs.* 3; column 5 *vs.* 7), indicating that silencing CHOP alone could not affect the cell viability. In the cells pre-treated with control siRNA, fucoidan treatment led to approximately 50–60% reduction of cell viability compared to those without fucoidan treatment (Fig. 7B, column 4 *vs.* 3; column 8 *vs.* 7; p<0.01). However, fucoidan treatment only caused approximately 10–20% reduction of cell viability in the CHOP-silenced cells (Fig. 7B, column 2 *vs.* 1; column 6 *vs.* 5). Of note, repression of CHOP in both types of cells led to a significant increase of cell viability after fucoidan treatment compared to the cells treated with control siRNA and fucoidan (Fig. 7B, column 2 *vs.* 4; column 6 *vs.* 8; p<0.05). Taken together, these results suggest that knockdown of CHOP could protect cells from fucoidan-induced cell apoptosis.

## Discussion

Fucoidan has been demonstrated to be a novel therapeutic agent for cancer treatment. Mechanistic studies revealed that fucoidan can suppress cancer cell survival, tumorigenesis and metastasis by modulating multiple signalling pathways [5], [7], [11]–[14]. In the present study, we demonstrate that fucoidan modulates ER stress cascades in cancer cells and the activated CHOP expression is responsible for fucoidan-induced cell apoptosis.

ER stress triggers a number of processes necessary for apoptosis. One of them is release of ER Ca^2+^ stores into the cytosol to activate the Ca^2+^-signal transducer CaMKII, leading to apoptosis through: (***i***) activation of c-Jun N terminal kinase (JNK) to induce death receptor Fas (11); (***ii***) stimulation of Ca^2+^ uptake by the mitochondria and release of apoptogens [36]. Hence, CaMKII activation by ER Ca^2+^ leak plays a crucial role in apoptosis. However, we observed that fucoidan treatment induced activation of p-CaMKII in MDA-MB-231 cells rather than in HCT116 cells. Consistently, the mitochondrial apoptotic protein Bax and the ER–associated caspase 12 were significantly increased in fucoidan-treated MDA-MB-231 cells. Considering that GRP78 can binds to ER Ca^2+^ and translocon in the ER membrane to block ER Ca^2+^ leak [18], this can be explained by the fact that fucoidan treatment in MDA-MB-231 cells, not in HCT116 cells, caused reduction of cytoprotective GRP78, thereby leading to ER Ca^2+^ leak into cytosol to activate CaMKII phosphorylation. In contrast, there was no significant change of CaMKII phosphorylation and Bax and caspase 12 expression in fucoidan-treated HCT116 cells, although CaMKII phosphorylation was moderately enhanced by fucoidan at 10 µg/ml and inhibited at 100 µg/ml. This is probably consistent with the observation that the expression of GRP78 was not significantly affected by fucoidan in HCT116 cells. However, the effect of fucoidan-induced downregulation of ERp29 on the moderate reduction of CaMKII phosphorylation in HCT116 cells should not be excluded. In addition, it has been reported that the ER-associated, Ca^2+^-induced caspase 12 activation is also implicated in ER stress-induced apoptosis [37], however, we did not observed activation of pro-caspase 12 (cleavage of caspase 12) in fucoidan-treated cells, suggesting that this caspase cascade may not be activated. Because GRP78 and ERp29 are essential ER proteins in maintaining the ER homeostasis and functions such as folding and secretion of proteins and degradation of misfolded proteins [29], [38], it is plausible that fucoidan potentiates damage to ER’s function through attenuating the expression of GRP78 or ERp29 in a cell context dependent manner, where the precise mechanisms need to be further explored.

ER stress-mediated signalling pathways are coupled to two main cascades: cell death-related PERK\P-eIF2α\CHOP cascade and cell survival-related AFT6 (IRE-1)\XBP-1 splicing cascade [17]. To cope with ER stress, XBP-1 mRNA is spliced by the activated p-IRE-1 to generate XBP-1s [32]. XBP-1s is a highly active transcription factor and is one of the key regulators of ER folding capacity [31]. Recent studies have shown that prolongation of IRE-1 signalling during ER stress can promote cell survival [39]. Hence, attenuation of this cascade could trigger cell apoptosis. Indeed, our studies demonstrated that fucoidan treatment significantly prevented XBP-1 splicing through inhibition of IRE-1 phosphorylation. On the other hand, our data also showed that fucoidan treatment could activate phosphotylation of eIF2α in both types of cells, similar to those observed in the cells treated with the inhibitor of eIF2α dephosphorylation, salubrinal. Moreover, these cells treated with salubrinal and fucoidan led to increased levels of p-eIF2α and CHOP over the cells treated with salubrinal alone. Although it is uncertain whether the fucoidan-induced increase of eIF2α phosphorylation is caused by the activated PERK in the ER or other kinases involved in the integrated stress response such as the general control nonrepressed 2 protein kinase and heme-regulated inhibitor [40], [41], cellular activation of p-eIF2α\CHOP by fucoidan potentially leads to cell apoptosis in these cancer cells. Recent study indicated a function of fucoidan in inducing ER stress-related autophagy and cell apoptosis in AGS cells [42]. It is thus suggested a central role of ER stress in fucoidan-induced cell apoptosis. Taken together, our studies reveal that fucoidan can broadly activate excessive ER stress cascades and subsequently induces cell apoptosis.

CHOP is a key regulator of ER stress-induced apoptosis. It regulates gene expression by forming hetero-dimers with other proteins from the C/EBP family or other transcription factors, acting as an activator or inhibitor of gene transcription [43]. In response to ER stress, DNA damage or other stress conditions, CHOP is induced to drive cell apoptosis by a variety of mechanisms including induction of oxidative stress and down-regulation of the anti-apoptotic protein Bcl2 [44]. The role of CHOP in fucoidan-induced cell apoptosis in MDA-MB-231 and HCT116 cells was verified by the fact that CHOP knockdown prevented DNA damage and cell death from fucoidan treatment. Therefore, the fucoidan-induced CHOP is responsible for fucoidan-induced cell apoptosis. Fucoidan treatment may result in the excessive phosphorylation of eIF2α to activate CHOP for the induction of cell apoptosis in cancer cells.

In summary, our studies provide definitive evidence that ER stress cascades play a critical role in fucoidan-induced cell apoptosis. Fucoidan broadly regulates ER stress by attenuating cell survival cascade and activating cell apoptotic cascade in cancer cells. The efficiency of fucoidan in killing cancer cells and preventing metastasis indicates its promising potential as a therapeutic agent in cancer treatment.

## Supporting Information

Table S1 **Summary of experimental data (mean±SD).**
(PDF)
