# Supplementary material for: Fucoidan Induces Cancer Cell Apoptosis by Modulating the Endoplasmic Reticulum Stress Cascades
Source: PLoS One. 2014 Sep 18;9(9):e108157. doi: 10.1371/journal.pone.0108157 (PMC4169461; doi:10.1371/journal.pone.0108157)
Supplement: Table S1 — Summary of experimental data (mean±SD). (PDF) [file pone.0108157.s001.pdf]

mean of band intensity of WB

231						
mean						
fucoidan	0	1	5	10	50	100
CHOP	0.9	1.2	1.5	1.65	1.6	1.78
P-eIF2a/eII	0.2	0.3	0.6	0.98	1	0.95
SD						
CHOP	0.12	0.06	0.1	0.1	0.05	0.12
P-eIF2a/eII	0.06	0.023	0.09	0.1	0.05	0.09
HCT						
	0	1	5	10	50	100
CHOP	0.4	0.6	0.7	1.2	1.26	1.6
P-eIF2a/eII	1	0.8	0.87	0.95	1.5	1.48
SD						
CHOP	0.05	0.1	0.08	0.13	0.09	0.12
P-eIF2a/eII	0.06	0.12	0.09	0.04	0.18	0.09
231						
mean						
	0	1	5	10	50	100
P-IRE1	0.65	0.6	0.51	0.4	0.24	0.15
XBP-1s	0.32	0.37	0.24	0.15	0.2	0.05
p58IPK	0.56	0.45	0.5	0.62	0.67	0.63
SD						
P-IRE1	0.05	0.06	0.08	0.04	0.05	0.04
XBP-1s	0.03	0.08	0.05	0.04	0.08	0.01
p58IPK	0.05	0.08	0.08	0.06	0.042	0.1
	0	1	5	10	50	100
HCT						
mean						
	0	1	5	10	50	100
P-IRE1	0.85	0.8	0.56	0.65	0.45	0.1
XBP-1s	1.2	1.32	0.95	0.52	0.51	0.12
p58IPK	0.48	0.4	0.45	0.38	0.42	0.46
sd						
	0.05	0.1	0.12	0.12	0.05	0.04
	0.1	0.05	0.09	0.1	0.08	0.01
	0.04	0.05	0.09	0.04	0.08	0.06

231

fucoidan	0	1	5	10	50	100
Bip	0.9	0.84	0.6	0.54	0.5	0.52
ERp29	0.34	0.25	0.3	0.34	0.31	0.28
SD						
BIP	0.05	0.09	0.05	0.04	0.05	0.06
ERp29	0.04	0.05	0.02	0.07	0.04	0.08

HCT

	0	1	5	10	50	100
Bip	0.75	0.8	0.89	0.78	0.6	0.7
ERp29	0.75	0.85	0.46	0.4	0.35	0.26
SD						
BIP	0.05	0.15	0.05	0.07	0.08	0.1
ERp29	0.1	0.15	0.02	0.07	0.04	0.035

231

MEAN	fucoidan	0	1	5	10	50	100
	Bax	0.2	0.8	1.1	1.4	1.2	1.5
	P-CaMKII/CaMI	0.75	1.03	1.2	1.4	2.08	2.16
	SD						
		0.056	0.05	0.12	0.08	0.13	0.09
		0.1	0.09	0.1	0.11	0.098	0.11

HCT

MEAN		0	1	5	10	50	100
	Bax	0.4	0.45	0.38	0.42	0.42	0.37
	P-CaMKII/CaMI	0.33	0.29	0.35	0.49	0.38	0.23
	SD						
		0.04	0.05	0.09	0.03	0.054	0.06
		0.03	0.06	0.09	0.04	0.034	0.03

Cas12

FUCOIDAN	MB-231	HCT116	sd		
0	0.2	0.12		0.06	0.04
10	0.36	0.08		0.045	0.06
50	0.78	0.15		0.08	0.056
100	0.82	0.2		0.056	0.034

cell viability

231

	sichop	sichop	sicontrol	sicontrol
fucoidan	0	100	0	100
mean	97	85	99	43
sd	8	4.5	2	5

hct

	sichop	sichop	sicontrol	sicontrol
fucoidan	0	100	0	100
mean	98	82	100	53
sd	6.5	7	3	7

cell growth

231

		mean			
days		1	2	3	4
Control		0.25	0.36	0.75	0.86
	10	0.24	0.34	0.62	0.76
	50	0.26	0.3	0.4	0.45
	100	0.23	0.26	0.36	0.36
sd		0.03	0.06	0.04	0.057
		0.03	0.07	0.06	0.05
		0.06	0.08	0.04	0.045
		0.06	0.056	0.034	0.065

cell growth

hct

		mean			
days		1	2	3	4
Control		0.25	0.6	0.84	0.95
	10	0.24	0.44	0.7	0.84
	50	0.26	0.3	0.43	0.54
	100	0.23	0.26	0.37	0.5
sd		0.03	0.06	0.04	0.057
		0.03	0.045	0.06	0.05
		0.06	0.08	0.04	0.045
		0.06	0.056	0.034	0.065

TUNEL

Control	Fucoidan	Control	Fucoidan	
2	25	3	32	mean
0.32	2.3	0.5	3.1	sd
